# Integrated untargeted and targeted metabolomics to reveal therapeutic effect and mechanism of *Alpiniae oxyphyllae* fructus on Alzheimer’s disease in APP/PS1 mice

**DOI:** 10.3389/fphar.2022.1104954

**Published:** 2023-01-11

**Authors:** Shengnan Zhou, Liwei Liu, Yuanyuan Zhang, Zhibo Zhang, Hanbing Li, Feng Fan, Jiuming He, Jian Kang, Lihua Zuo

**Affiliations:** ^1^ Department of Pharmacy, The First Affiliated Hospital of Zhengzhou University, Zhengzhou, Henan, China; ^2^ Henan Key Laboratory of Precision Clinical Pharmacy, Zhengzhou, Henan, China; ^3^ Henan Engineering Research Center of Clinical Mass Spectrometry for Precision Medicine, Zhengzhou, Henan, China; ^4^ College of Pharmacy, Henan University of Traditional Chinese Medicine, Zhengzhou, Henan, China; ^5^ Department of Neurointerventional radiology, The First Affiliated Hospital of Zhengzhou University, Zhengzhou, Henan, China; ^6^ State Key Laboratory of Bioactive Substance and Function of Natural Medicines, Institute of Materia Medica, Chinese Academy of Medical Sciences and Peking Union Medical College, Beijing, China

**Keywords:** Alpiniae oxyphyllae fructus, alzheimer’s disease, untargeted metabolomics, targeted metabolomics, bile acids

## Abstract

**Introduction:** *Alpiniae oxyphyllae* Fructus (AOF) has been abundantly utilized for the treatment of diarrhea, dyspepsia, kidney asthenia, and abdominal pain in China. AOF is effective for treating AD in clinical trials, but its exact mode of action is yet unknown.

**Methods:** In this study, metabolomics was combined to ascertain the alterations in plasma metabolism in APP/PS1 transgenic mice, the therapy of AOF on model mice, and the dynamic variations in 15 bile acids (BAs) concentration.

**Results:** 31 differential biomarkers were finally identified in APP/PS1 group vs. the WT group. The levels of 16 metabolites like sphinganine (Sa), lyso PE (20:2), lysoPC (17:0), glycocholic acid (GCA), deoxycholicacid (DCA) were increased in APP/PS1 group, and those of 15 metabolites like phytosphingosine, cer (d18:0/14:0), and fumaric acid were reduced in APP/PS1 group. After AOF treatment, 29 of the 31 differential metabolites showed a tendency to be back-regulated, and 15 metabolites were significantly back-regulated, including sphinganine (Sa), lyso PE (20:2), glycocholic acid (GCA), deoxycholic acid (DCA). The relationship between BAs level and AD had been received increasing attention in recent years, and we also found notable differences between DCA and GCA in different groups. Therefore, a BAs-targeted metabonomic way was established to determine the level of 15 bile acids in different groups. The consequence demonstrated that primary BAs (CA, CDCA) declined in APP/PS1 model mice. After 3 months of AOF administration, CA and CDCA levels showed an upward trend. Conjugated primary bile acids (TCA, GCA, TCDCA, GCDCA), and secondary bile acids (DCA, LCA, GDCA, TDCA, TLCA GLCA) ascended in APP/PS1 group. After 3 months of AOF treatment, the levels of most BAs decreased to varying degrees. Notably, the metabolic performance of DCA and GCA in different groups was consistent with the predictions of untargeted metabolomics, validating the correctness of untargeted metabolomics.

**Discussion:** According to metabolic pathways of regulated metabolites, it was prompted that AOF ameliorated the symptom of AD mice probably by regulating bile acids metabolism. This study offers a solid foundation for further research into the AOF mechanism for the therapy of AD.

## 1 Introduction

Alzheimer’s disease (AD) is a neurodegenerative illness with recollection loss and cognitive abnormalities as typical symptoms (Lane et al., 2018; Weller and Budson, 2018). In recent years, the number of AD patients and the fatality rate have risen year by year. It is challenging for ordinary households to afford the treatment of AD patients (Scheltens et al., 2021). Corresponding to this severe form, the drugs currently exploited to treat AD can only relieve the syndrome and cannot be cured (Li et al., 2021). The familiar pathological features of AD include abnormal accumulation of Aβ protein, hyperphosphorylation of tau protein, and synaptic dysfunction (Ju and Tam, 2022). The chemicals of traditional Chinese medicine (TCM) are plentiful and can act on multi-targets and multi-pathways (Li et al., 2021). In the treatment of AD, TCM has received more and more attention.

*Alpiniae oxyphyllae* fructus (AOF), which is the dry grown-up fruit of *Alpinia oxyphylla* Miq. Has a long history of application in traditional clinical Chinese medicine (Yang et al., 2020). In traditional medicine, AOF has been widely utilized for treatment of emesis, diarrhea, frequent urination, spermatorrhea and so on (Li R. et al., 2022). Modern pharmacological research manifested that AOF had anti-oxidant (Bian et al., 2013), anti-inflammatory (Qi et al., 2019), neuroprotective (Liu et al., 2020), anti-AD (Bian et al., 2021), and other effects. AOF contains a variety of chemical components, including sesquiterpenes, volatile oils, flavonoids, diarylheptanoids, steroids, and glycosides (Chen et al., 2013; Zhang et al., 2018). At present, the efficacy of AOF in AD is remarkable, but its potential therapeutic mechanism is not clear.

By using high-throughput data analysis, metabolomics seeks to keep track of how endogenous metabolites respond dynamically to external perturbations (Nielsen, 2017). Therefore, metabolomics is often used to observe the progression of diseases and to provide information on the relationship between diseases, drugs, and metabolites. Ultra high-performance liquid chromatography-quadrupole/orbitrap high-resolution mass spectrometry (UHPLC-Q-Orbitrap HRMS) is regarded as a suitable platform for metabolomic research. The mechanism of TCM therapy for AD had been extensively explained in recent years using untargeted metabolomics (Zhang et al., 2020; Chen et al., 2022; Wang et al., 2022). Targeted metabolomics characterize high sensitivity and quantitative precision, which focuses on a particular class of metabolites. In additon, the concept of the brain-gut axis has opened up a new perspective for the prevention and treatment of AD in recent years (Cryan et al., 2019; Doifode et al., 2021). Some scholars even claim that the gut was the “second brain”. The contact between the brain and the gut may be accomplished by BAs metabolism (Mulak, 2021). So, in comprehensive metabolomics, it is essential to pay attention to changes in typical BAs.

**FIGURE 1:**
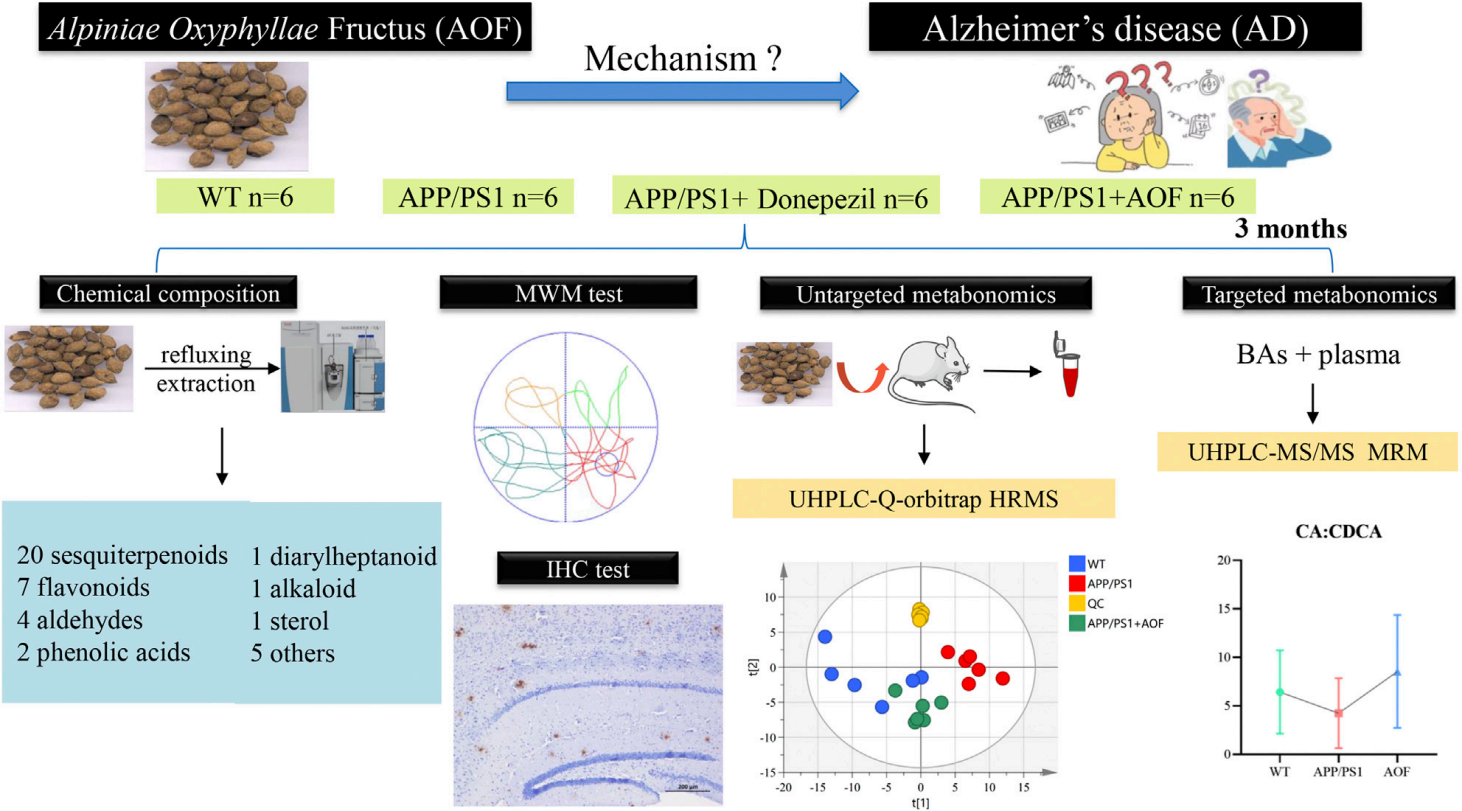
The flow chart of the whole experiment.

In the present study, the Morris Water Maze (MWM) test and immunohistochemical (IHC) test were utilized to evaluate the therapeutic effect of AOF on AD from behavioral and pathological perspectives, respectively. After that, the untargeted metabolomics based on UHPLC-Q-orbitrap HRMS discovered that the treatment of AD by AOF may be related to bile acid metabolism. In addition, 15 bile acids were quantified using targeted metabonomics analysis. The specific operation process of this study is shown in Figure 1. Untargeted and targeted metabolomics work together produced a highly accurate prediction of the mechanism of action of AOF in the treatment of AD. In conclusion, this study offeres additional proof that AOF can reduce AD symptoms, indicating that it may be used as a medicine to treat AD in the future.

## 2 Material and methods

### 2.1 Chemicals and materials

*Alpiniae Oxyphyllae* Fructus was obtained from the Henan Tongrentang Drug Co., Ltd. (Zhengzhou, China), which was authenticated by Professor Hanbing Li (Henan University of Chinese Medicine). The reference substances of oplopanone, nootkatone, and valencene were acquired from J&K Scientific Co., Ltd. (Beijing, China). The references of 15 bile acids and its isotope internal standard were purchased from Bazoe Medical Co., Ltd., containing cholic acid (CA), chenodeoxycholic acid (CDCA), taurocholic acid (TCA), glycocholic acid (GCA), taurochenodeoxycholic acid (TCDCA), glycoursodeoxycholic acid (GCDCA), deoxycholic acid (DCA), ursodeoxycholic acid (UDCA), lithocholic acid (LCA), taurodeoxycholic acid (TDCA), tauroursodeoxycholic acid (TUDCA), Glycoursodeoxycholic acid (GUDCA), Glycolithocholic acid (GLCA), taurolithocholic acid (TLCA), CA-d4, DCA-d4, LCA-d4, GLCA-d4, TCA-d5, TLCA-d5, TCDCA-d5 and GCA-d4. Purity of all standards is more than 98%. Donepezil hydrochloride were purchased from J&K Scientific Co., Ltd. (Beijing, China). The HPLC grade acetonitrile and methanol were obtained from Fisher Scientific (Pittsburgh, PA, United States of America). HPLC-grade formic acid was obtained from the Aladdin Industrial Corporation (Shanghai, China). There were also other analytical-grade chemicals and reagents.

### 2.2 Animals

APP/PS1 male mice (B6C3-Tg) and wild type (WT) littermates (both were 6 months old, 30–40 g in weight) were acquired from Huachuang Sino Pharmaceutical Technology Co., LTD. (Jiangsu, China). For 1 week before the experiment, all animals were kept at a constant temperature of 25°C, 65% humidity, and a 12-h light/dark cycle. After 7 days, the APP/PS1 mice were erratically separated into APP/PS1 (*n* = 6), APP/PS1+Donepezil (*n* = 6), APP/PS1+AOF (*n* = 6). The littermate wild-type mice served as the WT group (*n* = 6). Mice in the APP/PS1+Donepezil group were given donepezil orally (1 mg/kg) for 3 months. The APP/PS1+AOF group and was administrated with AOF at 0.5 g/kg for 3 months. In this work, the dosage was established using the dose conversion coefficient between mice and humans (Zuo et al., 2021). Saline solution (5 mL/kg/d) was intragastrically administered to the APP/PS1 group and the WT group for 3 months. All animal experimentation protocols adhered to the Experimental Animal Administration Regulations, and the operations were assented to by the Animal Ethics Committee of Zhengzhou University (No. ZZU-LAC20220225 [13]).

### 2.3 Preparation of AOF extract

AOF was elaborated by refluxing extraction way. The AOF was crushed and then passed through a 40-mesh sieve. 100 g AOF powder was precisely weighed, 10 times 95% ethanol was added, soaked for 1 h, and extracted at reflux for 1 h. The residue was extracted twice with 95% ethanol. Then the combined extracts were filtered, mixed, and dried under low pressure after being evaporated. The solution (1.0 g/mL) was made by adding methanol with the necessary amount of freeze-dried AOF powder and then put into an ultrasonic disperser for 10 min. The prepared solution was filtered by 0.22 μm membrane before entering to UHPLC-MS/MS system.

### 2.4 Morris water maze test

The MWM studies were conducted to assess the mice in each group’s capacity for spatial learning and memory following the final treatment (Li et al., 2022a). The experimental apparatus consists of a circular pool filled with opaque water (diameter 120, height 40), a circular target platform, and an image acquisition system. The target platform should be positioned in the middle of one of the pool’s four identically sized quadrants. Before the start of the experiment, opaque water 2 cm higher than the target platform was injected into the pool, and the water temperature was maintained at about 25°C ± 2°C. The MWM experiment is divided into two parts: the positioning and navigation test and the space exploration test. The day before the test, mice were arbitrarily swum in water for 3 min to adapt to the environment. The positioning and navigation experiment lasted for 5 days. Each mouse was placed in the water from two different quadrants every day, allowing it to freely find the target platform. Calculate the escape latency time by timing how long each mouse took to find the platform. On the sixth day, a space exploration experiment was carried out to investigate the memory ability of each group of mice. The platform was removed and the mice were forced to swim from two different quadrants. The time spent in the target quadrant and crossing times were recorded and analyzed by the SANS video tracking system (SA201, Jiangsu, China). Continuous intragastric administration during this experiment. Finally, all experimental data of MWM were statistically analyzed by GraphPad Prism 9.4.1 software. The Student’s t-test was employed to compare the two groups, while the ANOVA was utilized to compare multiple groups. All data were displayed in means ± standard deviation (Mean ± SD). *p* < 0.05 was treated as a statistically observable difference.

### 2.5 Sample collection

The animals underwent a one-night fast, anesthesia, heart puncture blood collection, and 5% sodium heparin anticoagulation after the MWM experiment. After centrifuging at 3500 rpm for 10 min by 4°C centrifuge, the supernatant plasma was transferred for metabolomic analysis. After the blood is taken, the skull is opened and the whole brain is removed, immersed in 4% paraformaldehyde (PFA) for further immunohistochemistry (IHC) assay.

### 2.6 Immunohistochemistry

Immunohistochemistry (IHC) was used to assess pathological alterations in the mouse’s brain’s cortex and hippocampus. The brain tissue was sectioned and fixed in paraffin. Then gradually started the following operations. 1) Paraffin sections were dewaxed and hydrated by xylene and gradient ethanol. 2) To repair the antigens, the sections were submerged in citric acid (pH 6.0) antigen repair solution. 3) 3% H_2_O_2_ was added to block endogenous peroxidase. 4) The tissue was uniformly covered with 3% BSA in the tissue ring and closed at room temperature for 30min. 5) Add 1:500 diluted rabbit anti-beta-amyloid dropwise. 6) Slides were washed 3 times with shaking in PBS (pH 7.4) for 5 min each time. Subsequently, goat anti-rabbit IgG (1:200, HRP labeled) was added to the circle, and incubate at room temperature for 50min. 7) Add DAB coloring solution, positive for brownish yellow. 8) The cell nucleus was restained with hematoxylin and dehydrated. 9) Microscopic examination, image collection, and analysis.

### 2.7 Plasma untargeted metabolomics

#### 2.7.1 UHPLC-Q-orbitrap HRMS method

In the qualitative profiling of AOF extracts and plasma untargeted metabolomics, an UHPLC-Q-orbitrap HRMS system was utilized. The column adopted a Waters ACQUITY UPLC HSS T3 column (2.1 × 100 mm, 1.8 µm) adhered to 40°C of the column oven temperature. The mobile phase consisted of pure water with 0.1% (v/v) formic acid A) and acetonitrile B). The gradient elution was set as follows: For qualitative profiling of AOF extracts 0–3.0 min, 5% B; 3.0–35 min, 5.0%–25% B; 35–43 min, 25%–40% B; 43–53 min, 40%–100% B;53–57 min, 100% B; 57.1–60 min, 5% B; For plasma untargeted metabolomics: 0–1.0 min, 5% B; 1.0–9.0 min, 5%–100% B; 9.0–12.0 min, 100% B, 12.0–12.1, 100%–5% B, 12.1–15 min, 5% B; The flow rate and injection volume were 0.3 mL/min and 5 μl, respectively.

The optimized MS function was as follows: ion spray voltage, +3.5 kV and −2.8 kV; the ion source temperature was 350°C; the capillary mperature was 320°C; auxiliary gas flow rate, 10 arb; the sheath gas flow rate, +40 arb and -38 arb; the scan range was from 80 to 1200 m*/z* in all ion modes. The resolution of full MS scan and dd-MS^2^ scan were 70,000 and 17,500, respecively. Nitrogen as the collision gas was used to stabilize the spray.

#### 2.7.2 Blood sample preparation

The plasma samples were taken out of the −80°C refrigerator and melted on ice. The 150 μl aliquot of ice-cold methanol (containing 500 ng/mL, ketoprofen +50 ng/mL, 2-chloro-L-phenylalanine) was deposited with a 50 µl plasma sample. Then the mixture was vortexed for 1 min and centrifuged for 10 min at 13,300 rpm. Finally, transferred the supernatant to the sample vial. The order of injection of samples was random. The QC sample was collected from an equal volume of each plasma sample. And every five sample runs in the analytical batch, a QC sample was examined to evaluate the robustness of the analytical platform.

#### 2.7.3 Data processing and statistical analysis

For the untargeted metabolomic analysis, UHPLC-Q-orbitrap HRMS data was processed by Compound Discoverer (CD) 3.3 software for peak alignment. CD provided a data matrix containing retention time, accurate relative molecular mass, and peak area of each compound. Subsequently, this data matrix was used for multivariate statistical analysis by SIMICA software (version 14.1, Umetrics, Sweden). To understand the changes in overall metabolism, evaluate the stability of QC samples, and filtered out the outliers, PCA analysis was used to process the data matrix. The OPLS-DA was used to specifically observe the separation between the two groups. 200 permutations test was utilized to appraise the validity of the OPLS-DA model. The features with variable importance in the projection (VIP) > 1.0 and *p*-value <0.05 were deemed as potential differential metabolites.

### 2.8 Targeted metabolomics analysis of bile acids

#### 2.8.1 UHPLC-MS/MS -MRM method

In the quantitative analysis of fifteen bile acids, an UHPLC-MS/MS system was employed. Chromatographic separation occurred in a Waters ACQUITY UPLC^®^ BEH C_18_ column (2.1 mm × 50 mm, 1.7 μm). The flow rate was 0.6 mL/min. A (water) and a mixture of B (acetonitrile containing 2% water, 0.2% ammonium formate (1 mol/L), and 0.05% ammonia water) made up the mobile phase. The gradient elution procedure was as follows: 30% B (0–0.6 min), 30%–40% B (0.6–2.6 min), 40%–95% B (2.6–3.0 min), 95% B (3.0–3.5 min), 95%–24.5% B (3.5–3.8 min), and continuing at 24.5% B up to 7.0 min. For all samples and standard solutions, the injection volume was 5 μl.

The AB SCIEX™ × 3200 spectrometer (Foster City, CA) equipped with a Shimadzu Prominence UHPLC (Pleasanton, CA). The mass spectrometer was run in the ESI^−^ mode with multiple reaction monitoring (MRM). The column temperature and sample tray temperature were 55°C and 8°C. The parameters were as follows: ion spray voltage was set at –4.5 kV, source temperature maintained at 500°C, and the flow of ion source gas1 (GS1) and ion source gas2 (GS2) were 40 L/h and 60 L/h, respectively. The transitions and collision energy levels of 15 BAs are lsited in Supplementary Table S2.

#### 2.8.2 Standard stock solutions preparation

Each bile acid was individually dissolved in the suitable ratio of 50% methanol to produce the stock standard solutions (1.0 mg/mL). The final mixed standard solution was created by combining each stock solution with 50% methanol. The mixture of standard solutions was successively diluted with 50% methanol to generate several working standard solutions. The middle solution was then constantly diluted in 50% acetonitrile to obtain working solutions involving 80, 160, 320, 640, 1280, 2560, and 5120 ng/mL of CA, DCA, CDCA, LCA, GCA, GLCA, GCDCA, TLCA, TDCA, TCDCA, TUDCA; Low, medium and high concentrations of these acids were 480, 960, and 1920 ng/mL 40, 80, 160, 320, 640, 1280, and 2560 ng/mL of GUDCA, UDCA, GDCA, and TCA; Low, medium, and high concentrations of these bile acids were 240, 480, and 960 ng/mL.

#### 2.8.3 Data acquisition and processing

The Analyst^®^ 1.6.2 application (AB SCIEX™, Foster City, CA) was used for acquiring and integrating data. The software allows the exported data to be saved in the proper folder.

## 3 Results

### 3.1 Identification of the chemical composition of AOF

The base peak intensity (BPI) chromatograms demonstrate in Supplementary Figure S1. A total of 41 compounds were identified from AOF (Supplementary Table S1), including 20 sesquiterpenoids, seven flavonoids, 4 aldehydes, 2 phenolic acids, 1 diarylheptanoid, 1 alkaloid, 1 sterol, and 5 others. As the main characteristic chemical components of AOF, sesquiterpenoids are closely related to the neuroprotective effect. The 20 sesquiterpenoids found in this study included seven eudesmane sesquiterpenoids, seven eremophilane sesquiterpenoids, 5 cadinane sesquiterpenoids, and 1 oplopanone sesquiterpenoids.

### 3.2 AOF improves learning and memorizing abilities of APP/PS1 model mice

**FIGURE 2:**
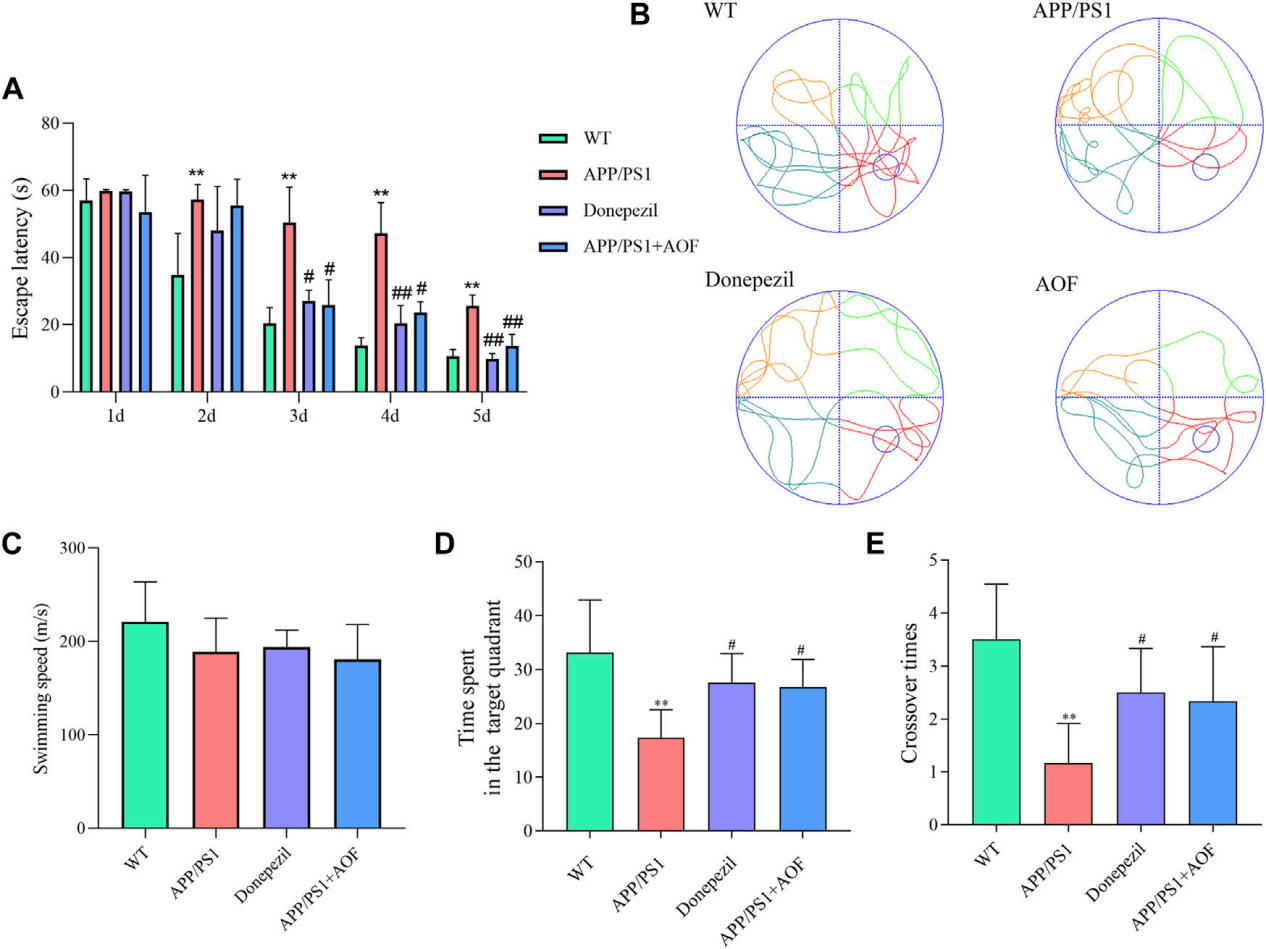
Effect of AOF on APP/PS1 mice in MWM. **(A)** Escape latency in directional navigation experiment. **(B)** Representative images of swimming trajectories. **(C)** The swimming speeds of mice in different groups **(D)** Time in the target quadrant in spatial probe task. **(E)** Number of platform crossings in spatial probe task. ^*^ *p* < 0.05, ^**^ *p* < 0.01, compared with WT group; ^#^ *p* < 0.05, ^##^ *p* < 0.01, compared with APP/PS1 group.

To investigate the influence of AOF on learning and memorizing abilities in AD mice, MWM was carried out 3 months after administration. As shown in Figure 2A, in the previous directional navigation experiment, with the extension of training days, the escape latency of all groups gradually declines. In addition, from the day 2, the escape latency of the APP/PS1 model group was longer than that of the WT group (*p* < 0.01).

On the day 3, compared with APP/PS1 model group, the escape latency of the APP/PS1+Donepezil group and APP/PS1+AOF began to decrease (*p* < 0.05). The escape latency of the APP/PS1+Donepezil group significantly decreased on days 4 and 5 of treatment (*p* < 0.01). Although the effect of AOF on the escape latency was not as fast as the positive drug, but the escape latency of APP/PS1+AOF was also observably decreased on the day 5 (*p* < 0.01). As shown in Figure 2B **,** the swimming trajectories of the WT group were complex, but the APP/PS1 group was single and clear. There was no difference in average swimming speed among all groups (Figure 2C). In the spatial probe task, compared with the WT group, the time spent in the target quadrant and the number of platform crossings of the APP/PS1 model group were markedly reduced (*p* < 0.01). After AOF treatment, both indicators of model mice were improved (*p* < 0.05). To sum up, these results proved that oral AOF can alleviate cognitive impairment in APP/PS1 mice.

### 3.3 AOF relieves the Aβ deposition in the brain of APP/PS1 mice

**FIGURE 3:**
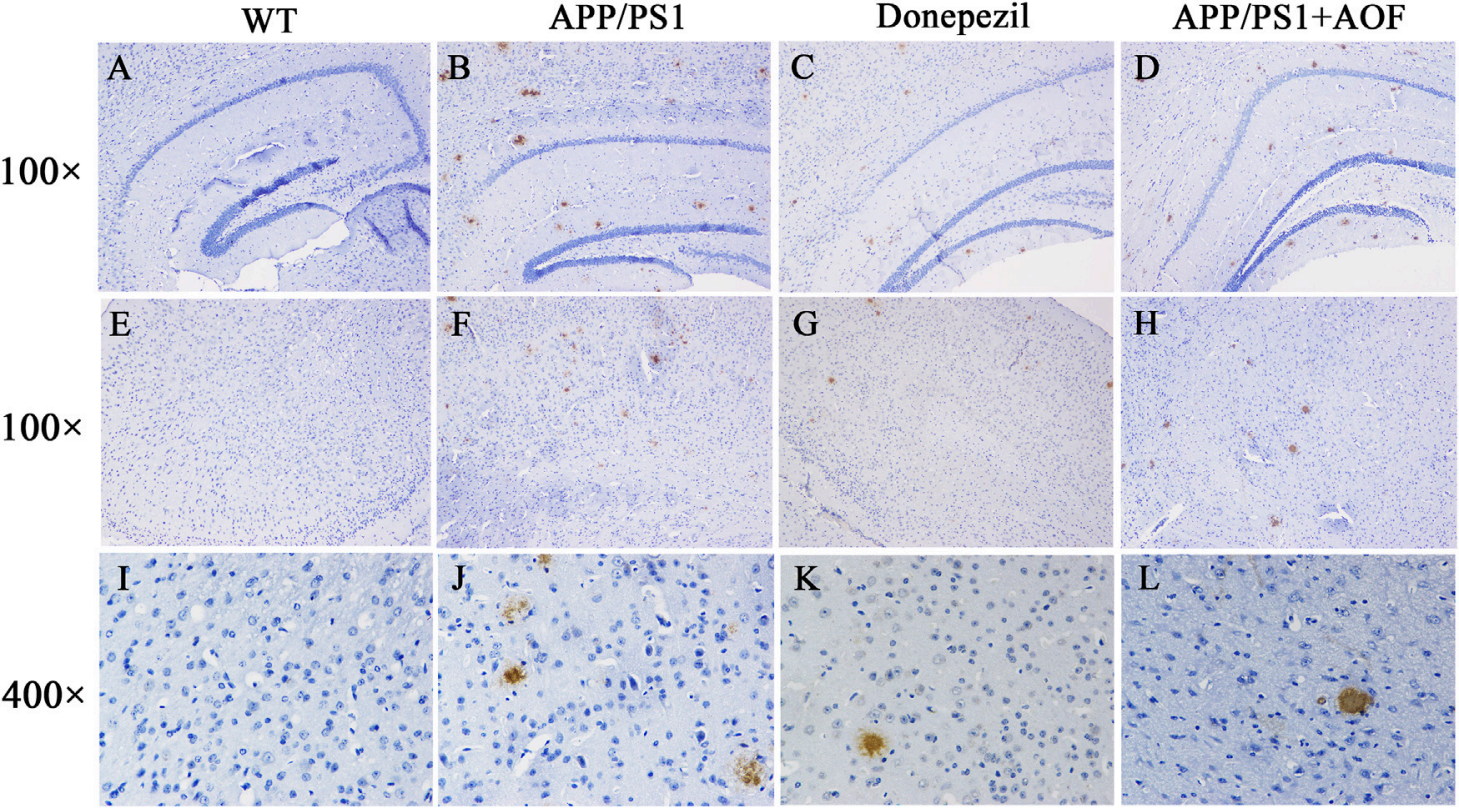
Effect of AOF on APP/PS1 mice in IHC test. **(A–D)** DAB-stained Aβ plaques in the hippocampal region (100×) of WT, APP/PS1, APP/PS1+ Donepezil, APP/PS1+AOF groups. **(E–H)** DAB-stained Aβ plaques in the cortex (100×) of WT, APP/PS1, APP/PS1+ Donepezil, APP/PS1+AOF groups. **(I–L)** Amplified views (400×) of the cortex in each group. WT, APP/PS1, APP/PS1+ Donepezil, APP/PS1+AOF groups.

After the MWM experiment, all brain tissues were collected for the IHC test. The expressions of Aβ proteins in the hippocampus and cerebral cortex of 4 groups were observed and compared by IHC. As shown in Figure 3 (AB, DE, IJ), compared with the WT group, Aβ plaques were clearly increased in the hippocampus and cortex of the APP/PS1 group. After AOF treatment in 3 months, The Aβ plaques of brain tissue were significantly reduced Figure 3 (BD, FH, JL), displaying an alike result as the mice of APP/PS1+Donepezil group.

### 3.4 Untargeted plasma metabonomics results

#### 3.4.1 Multivariate statistical analysis of plasma metabolites

**FIGURE 4:**
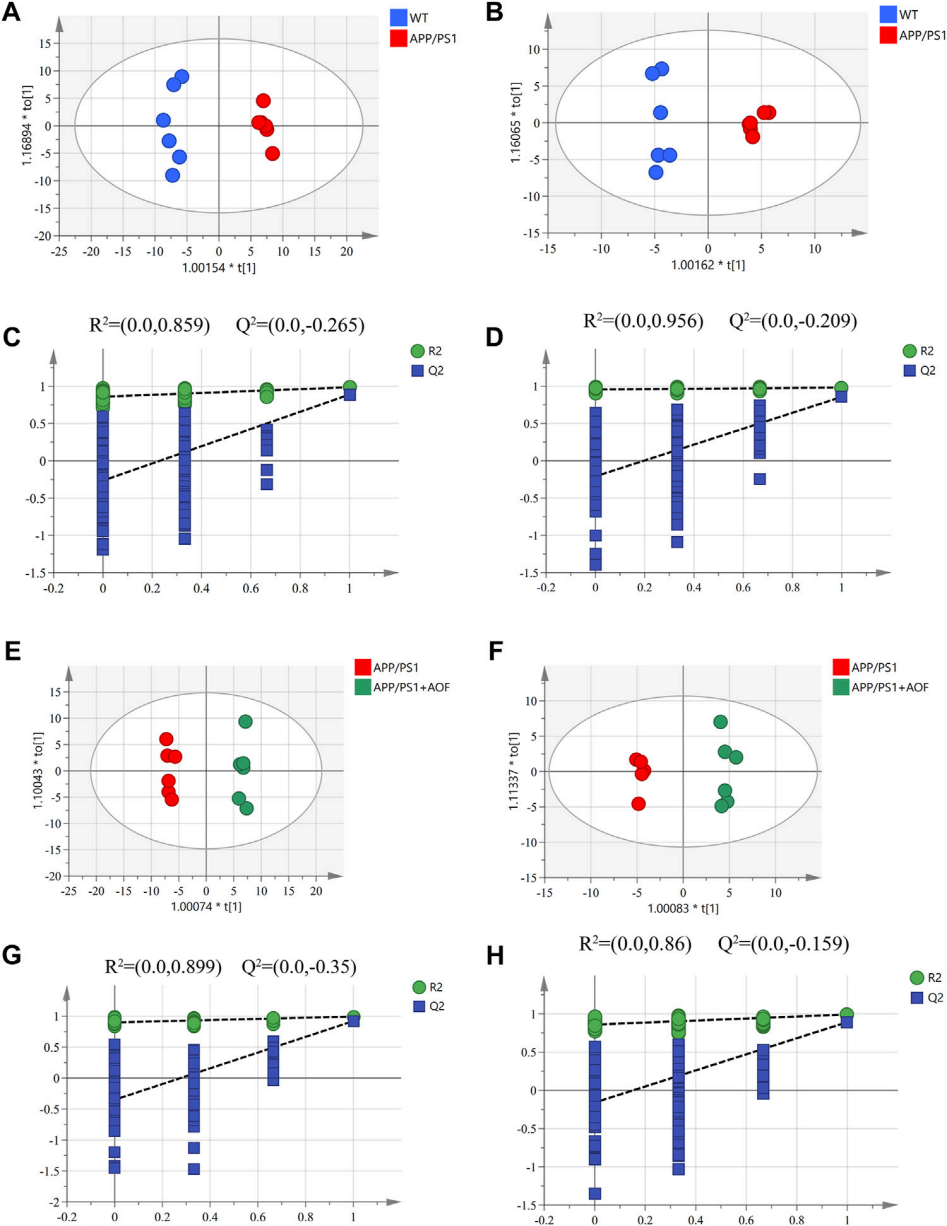
Multivariate statistical analysis of the plasma metabolites. (**A–B**) OPLS-DA score plots for APP/PS1 vs. WT in ESI^+^ and ESI^−^ ion mode. (**C–D**) 200 permutation tests for APP/PS1 vs. WT in ESI^+^ and ESI^−^ ion mode. (**E–F**) OPLS-DA score plots for APP/PS1+AOF vs*.* APP/PS1 in ESI^+^ and ESI^−^ ion mode. (**G–H**) 200 permutation tests for APP/PS1+AOF vs*.* APP/PS1 in ESI^+^ and ESI^−^ ion mode.

UHPLC-Q-Orbitrap HRMS was utilized to explore the plasma metabolic profiles of WT, APP/PS1, and AOF mice in ESI^+^ and ESI^−^ modes. To study the changes in the overall metabolism of each group, unsupervised PCA analysis was firstly used to evaluate the mass spectrometry data. As shown inSupplementary Figure S2 **,** the WT group, APP/PS1 group, and AOF group showed a significant tendency of separation. The QC samples are clustered together, which indicates the stability of the analytical instrument. Then, we used OPLS-DA to specifically observe the separation between the two groups. As shown in an OPLS-DA score, there was a clear separation in APP/PS1 group vs*.* WT group (Figures 4A,B) and AOF group vs*.* APP/PS1 group (Figures 4E,F). In addition, 200 permutation tests were utilized to check the above OPLS-DA model, and the outcomes demonstrate that the model has an excellent fitting degree and excellent prediction ability (Figures 4C,D; Figure 4 G, H).

#### 3.4.2 Differential metabolite identification and analysis

With the UHPLC-Q-Orbitrap HRMS metabolomics platform, 115 ion peaks were found in APP/PS1 group vs*.* the WT group by VIP values >1.0 and *p* values <0.05. Subsequently, 31 endogenous differential metabolites were defined in APP/PS1 group vs*.* WT group (Table 1). The levels of 16 metabolites like sphinganine, and lyso PE (20:2), lysoPC(17:0), glycocholic acid (GCA), deoxycholic acid (DCA) were increased in APP/PS1 group, and those of 15 metabolites like phytosphingosine, cer (d18:0/14:0), and fumaric acid were reduced in APP/PS1 group. As shown in Table 1, when AOF was administered for 3 months, 94% (29/31) of the metabolites had a tendency to retract and 48% (15/31) had a significant retract (*p* < 0.05).

**TABLE 1:** Differential metabolites between APP/PS1 vs*.* WT and the callback function of AOF.

Ion mode	Metabolite	Formula	ppm	*m/z*	Rt	APP/PS1/WT	AOF/A*p*P/PS1
ESI+	3-Oxalomalic acid	C_6_H_6_O_8_	2.44	206.0068	0.809	↑*	↓
	Aminoadipic acid	C_6_H_11_NO_4_	−0.16	161.0688	0.868	↓*	↑
	N6-Acetyl-L-lysine	C_8_H_16_N_2_O_3_	−1.24	188.1159	1.272	↓*	↑
	Nicotinic acid	C_6_H_5_NO_2_	0.58	123.0321	1.273	↓*	↑^#^
	Adenine	C_5_H_5_N_5_	2.17	135.0548	1.329	↓*	↑^#^
	Adenosine	C_10_H_13_N_5_O_4_	−1.13	267.0965	1.331	↓*	↑^#^
	Aspartyl-Leucine	C_10_H_18_N_2_O_5_	−0.52	246.12144	2.258	↓*	↑
	Phytosphingosine	C_18_H_39_NO_3_	-2.41	317.2922	7.258	↓*	↑
	Sphinganine	C_18_H_39_NO_2_	−2.29	301.2974	7.565	↑*	↓^##^
	Sphinganine 1-phosphate	C_18_H_40_NO_5_P	−1.97	381.2637	7.811	↑**	↓
	LysoPE (20:2)	C_25_H_48_NO_7_P	1.61	505.3177	8.383	↑*	↓^##^
	LysoPC(17:0)	C_25_H_52_NO_7_P	−0.52	509.3479	9.667	↑*	↓^#^
	Cer(d18:0/14:0)	C_32_H_65_NO_3_	−1.34	511.4958	9.675	↓*	↑
	Linoleamide	C_18_H_33_NO	−1.95	279.2557	9.784	↑**	↓^##^
	Oleamide	C_18_H_35_NO	−1.99	281.2713	10.35	↑*	↓^##^
	Hexadecanamide	C_16_H_33_NO	−2.05	255.2557	11.103	↓*	↓
ESI-	Fumaric acid	C_4_H_4_O_4_	−10.46	116.0097	0.929	↓*	↑^#^
	N-Acetyl-L-glutamic acid	C_7_H_11_NO_5_	−4.16	189.0629	1.363	↓*	↑
	Tyrosine	C_9_H_11_NO_3_	−4.77	181.073	1.383	↓*	↓
	2-methylcitric acid	C_7_H_10_O_7_	−2.98	206.042	1.392	↓*	↑
	L-gamma-Glutamyl-L-leucine	C_11_H_20_N_2_O_5_	−0.47	260.1371	3.565	↓*	↑
	Thromboxane B2	C_20_H_34_O_6_	−0.01	370.2355	6.086	↑*	↓^#^
	Glycocholic acid	C_26_H_43_NO_6_	−0.27	465.3089	6.369	↑*	↓^##^
	Sphingosine 1-phosphate	C_18_H_38_NO_5_P	−0.15	379.2487	7.642	↑**	↓^#^
	LysoPE (22:6)	C_27_H_44_NO_7_P	−1.09	525.285	8.189	↑*	↓^#^
	Docosahexaenoic Acid	C_22_H_32_O_2_	−1.01	328.2399	8.204	↑*	↓^#^
	Deoxycholic acid	C_24_H_40_O_4_	-0.66	392.2924	8.259	↑*	↓^##^
	Palmitic acid	C_16_H_32_O_2_	−1.94	256.2397	8.392	↑**	↓
	Stearic acid	C_18_H_36_O_2_	−1.16	284.2712	9.306	↑*	↓
	Eicosapentanoic acid	C_20_H_30_O_2_	−0.32	302.2245	9.851	↑**	↓
	Tiaprost	C_20_H_28_O_6_S	−1.44	396.1601	10.383	↓*	↑

**FIGURE 5:**
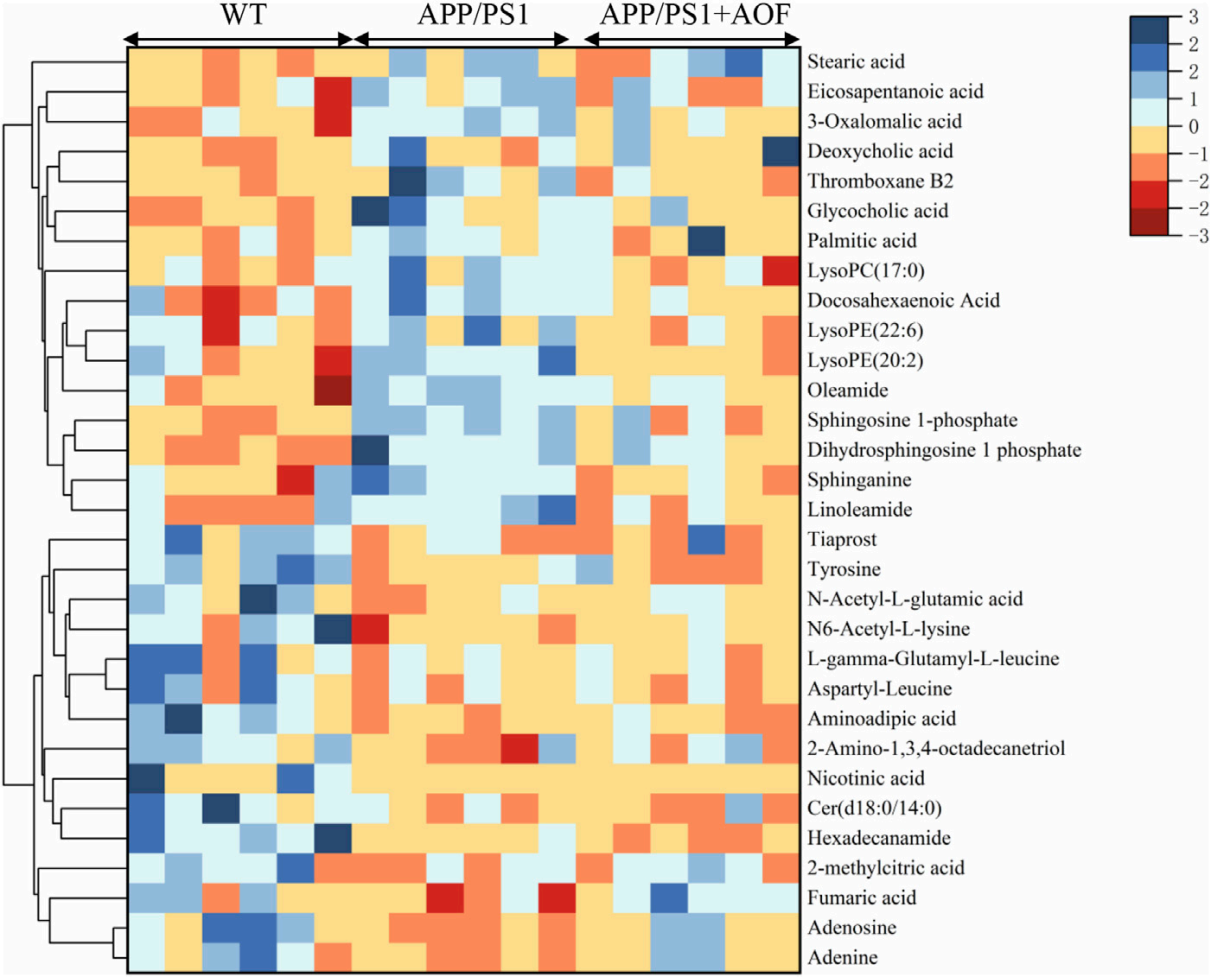
Heat map of differential metabolites in the plasma.

To more vividly show the changes of metabolites in plasma between different groups, Origin 2021 was used for heat mapping (Figure 5). The difference between the groups is obvious according to the color. Compared with the WT group, the metabolites of the APP/PS1 group displayed a different color trend. It is worth noting that most of the dysregulated metabolites were improved after the administration of AOF. The above differential metabolite results indicated that AOF could reverse the metabolic abnormalities in model mice.

**FIGURE 6:**
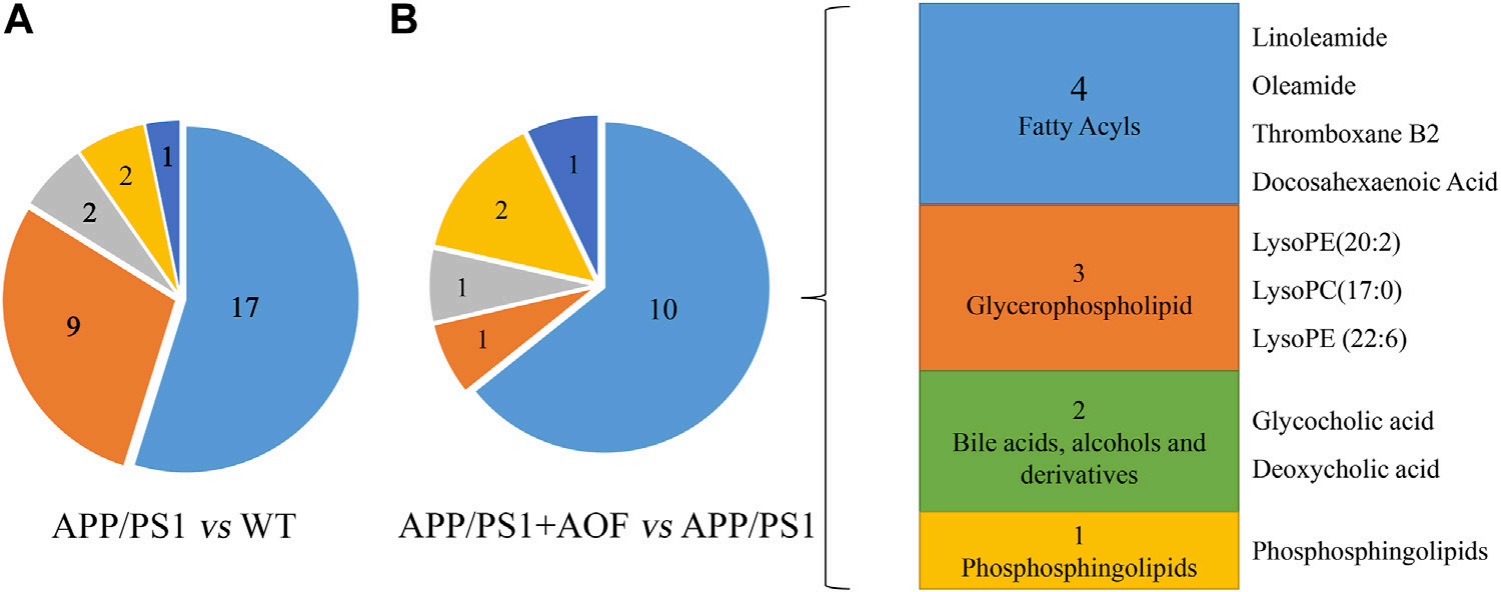
Differential metabolites were classified by HMDB database. **(A)** Differential metabolites were classified for APP/PS1 vs. WT. **(B)** Differential metabolites were classified for APP/PS1+AOF vs*.* APP/PS1.

Furthermore, the differential metabolites were classified by HMDB. The classification results are displayed in pie charts. As shown in Figure 6A and Table 2, the different colors and areas represent different HMDB classifications and metabolite numbers. The results demonstrated that the 31 differential metabolites included 17 lipids and lipid-like molecules, 9 organic acids and derivatives, 2 organoheterocyclic compounds, 2 organic nitrogen compounds, 1 nucleosides, nucleotides, and analogues. The pie chart of the 15 metabolites significantly regulated by AOF was shown in Figure 6B. Of the 15 metabolites significantly regulated by AOF, 2 metabolites (DCA, GCA) were classified as bile acids, alcohols and derivatives.

**TABLE 2:** Detailed classification of differential metabolites.

A		
Linoleamide	Lipids and lipid-like molecules	Fatty Acyls
Oleamide	Lipids and lipid-like molecules	Fatty Acyls
Hexadecanamide	Lipids and lipid-like molecules	Fatty Acyls
Thromboxane B2	Lipids and lipid-like molecules	Fatty Acyls
Docosahexaenoic Acid	Lipids and lipid-like molecules	Fatty Acyls
Palmitic acid	Lipids and lipid-like molecules	Fatty Acyls
Stearic acid	Lipids and lipid-like molecules	Fatty Acyls
Eicosapentanoic acid	Lipids and lipid-like molecules	Fatty Acyls
Tiaprost	Lipids and lipid-like molecules	Fatty Acyls
LysoPE (20:2)	Lipids and lipid-like molecules	Glycerophospholipid
LysoPC(17:0/0:0)	Lipids and lipid-like molecules	Glycerophospholipid
LysoPE (22:6)	Lipids and lipid-like molecules	Glycerophospholipid
Glycocholic acid	Lipids and lipid-like molecules	Bile acids, alcohols and derivatives
Deoxycholic acid	Lipids and lipid-like molecules	Bile acids, alcohols and derivatives
Sphingosine 1-phosphate	Lipids and lipid-like molecules	Phosphosphingolipids
Dihydrosphingosine 1 phosphate	Lipids and lipid-like molecules	Phosphosphingolipids
Cer(d18:0/14:0)	Lipids and lipid-like molecules	Ceramides
B		
3-Oxalomalic acid	Organic acids and derivatives	Carboxylic acids and derivatives
L-gamma-Glutamyl-L-leucine	Organic acids and derivatives	Carboxylic acids and derivatives
Aminoadipic acid	Organic acids and derivatives	Carboxylic acids and derivatives
N6-Acetyl-L-lysine	Organic acids and derivatives	Carboxylic acids and derivatives
Aspartyl-Leucine	Organic acids and derivatives	Carboxylic acids and derivatives
2-methylcitric acid	Organic acids and derivatives	Carboxylic acids and derivatives
Fumaric acid	Organic acids and derivatives	Carboxylic acids and derivatives
N-Acetyl-L-glutamic acid	Organic acids and derivatives	Amino acids, peptides, and analogues
Tyrosine	Organic acids and derivatives	Amino acids, peptides, and analogues
C		
Sphinganine	Organic nitrogen compounds	Amines
Phytosphingosine	Organic nitrogen compounds	Amines
D		
Nicotinic acid	Organoheterocyclic compounds	Pyridinecarboxylic acids and derivatives
Adenine	Organoheterocyclic compounds	Purines and purine derivatives
E		
Adenosine	Nucleosides, nucleotides, and analogues	Purine nucleosides

#### 3.4.3 Metabolic pathway analysis

**FIGURE 7:**
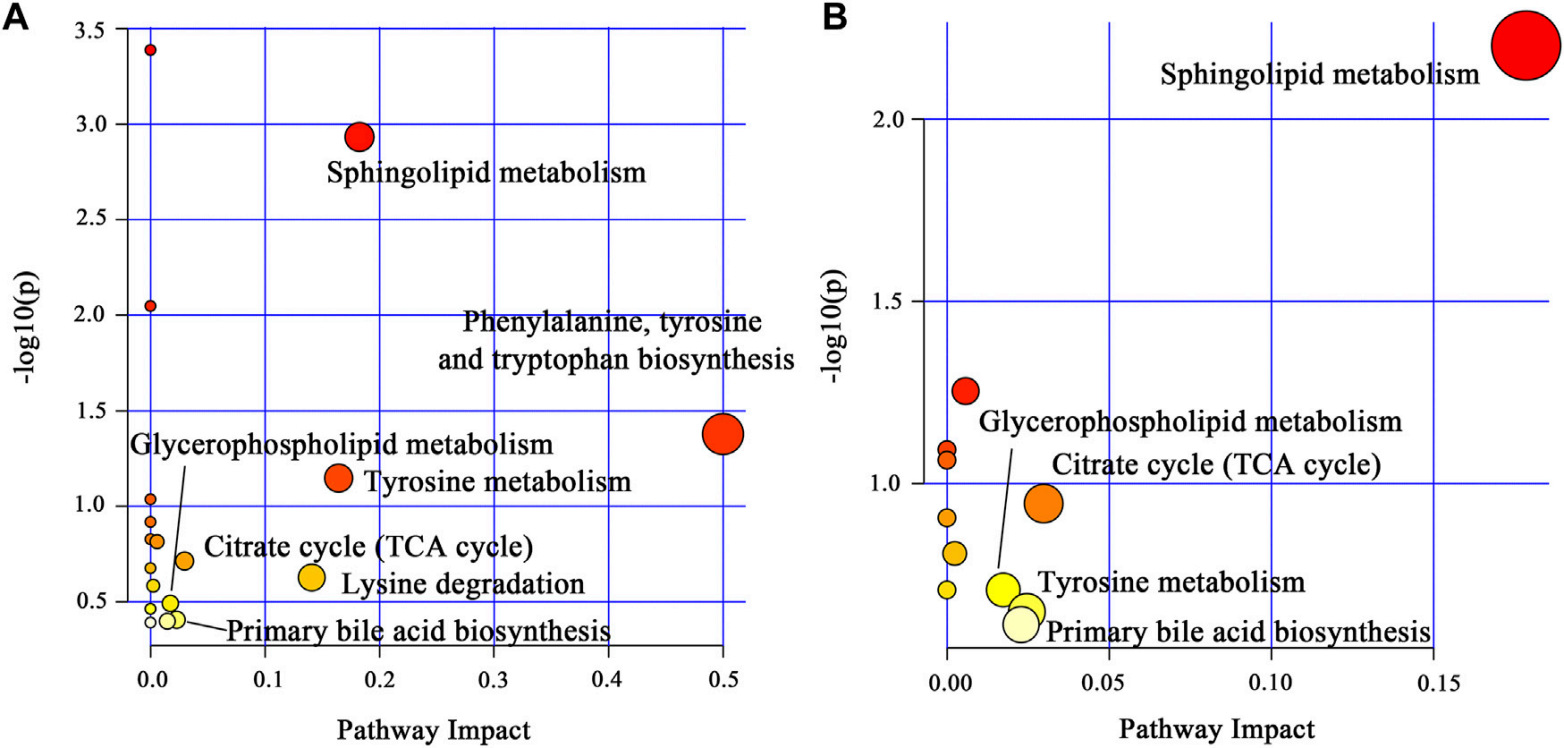
**(A)** Main metabolic pathways of the differential metabolites for APP/PS1 vs. WT. **(B)** Main metabolic pathways of the differential metabolites for APP/PS1+AOF vs*.* APP/PS1.

**FIGURE 8:**
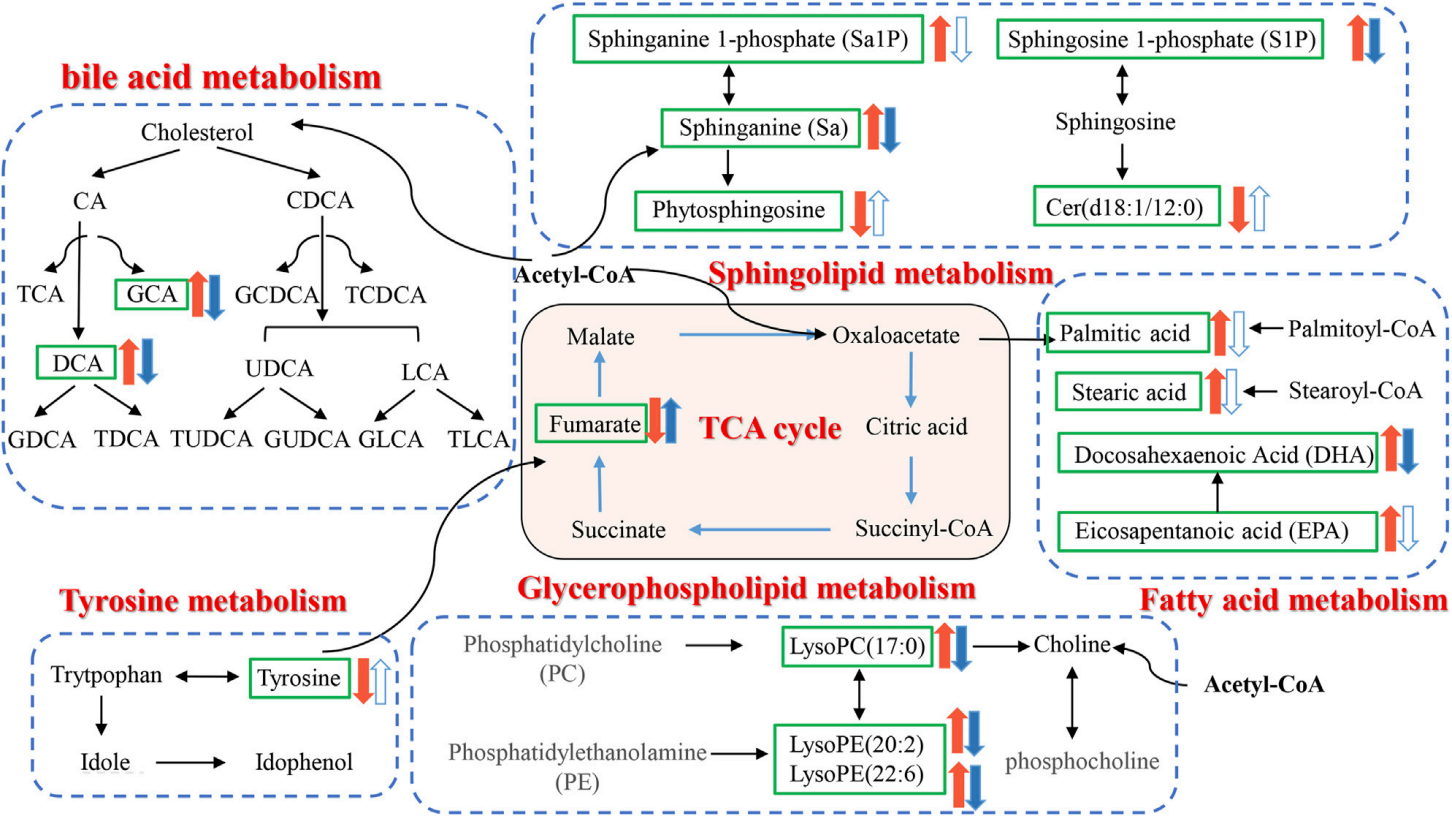
Disorder metabolic network in APP/PS1 mice and the interventional effect of AOF.

To explore the potential mechanism of AOF in alleviating AD symptoms, the differential metabolites in each group were introduced into Metabo Analyst 5.0 for further biological analysis. The 31 differential metabolites in APP/PS1 vs*.* WT were mainly enriched in sphingolipid metabolism, phenylalanine, tyrosine and tryptophan biosynthesis, tyrosine metabolism, TCA cycle, lysine degradation, glycerophospholipid metabolism, primary bile acid biosynthesis (Figure 7A). Compared with the APP/PS1 group, significantly callback metabolites in the APP/PS1+AOF group were mainly participate in sphingolipid metabolism, TCA cycle, glycerophospholipid metabolism, tyrosine metabolism, and primary bile acid biosynthesis (Figure 7B). Subsequently, the information on differential metabolites and metabolic pathways was integrated to construct a comprehensive network metabolic map for the AOF treatment of AD (Figure 8). This figure clearly shows the changes and associations of various metabolites in APP/PS1 vs*.* WT, and APP/PS1+AOF vs*.* APP/PS1. It should be noted that the metabolic disorders and the improvement of AOF were closely related to the metabolic pathway of BAs.

### 3.5 Targeted metabolomics

To assess the intimacy between changes in BAs metabolism and the relief of AD symptoms by AOF, we performed targeted quantitative validation of the bile acid profile by UHPLC-MS/MS-MRM method. Primary BAs (CA, CDCA) come from cholesterol in the liver. GCA and TCA are glycine-conjugate and taurine-conjugate of CA respectively. Similarly, GCDCA and TCDCA are glycine-conjugate and taurine-conjugate of CDCA. Under the action of anaerobic bacteria, CA is converted to secondary bile acids DCA, and CDCA is converted to secondary bile acids LCA and UDCA. DCA, UDCA, and LCA are coupled with glycine and taurine to form GDCA, TDCA, GUDCA, TUDCA, GLCA, and TLCA, respectively.

**FIGURE 9:**
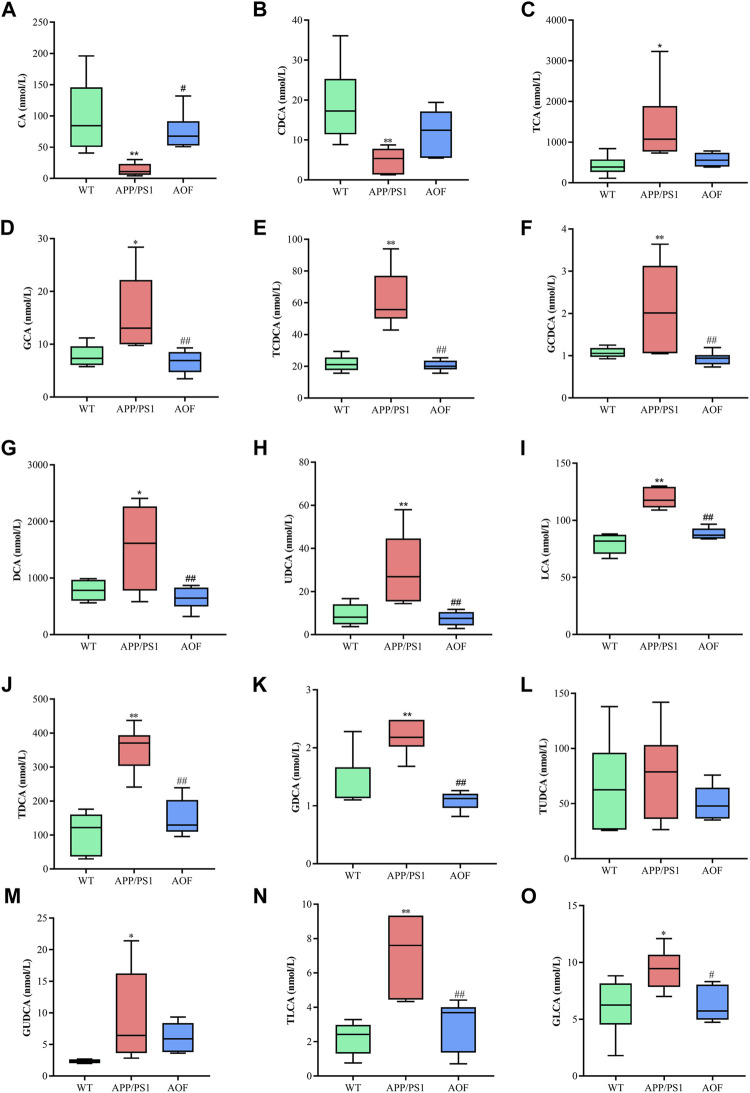
AOF reversed most BAs levels in different groups of APP/PS1 mice. **(A–O)** CA, CDCA, TCA, GCA, TCDCA, GCDCA, DCA, UDCA, LCA, TDCA, GDCA, TUDCA, GUDCA, TLCA, and GLCA, respectively. Data are presented as mean ± SEM (*n* = 6). ^*^ *p* < 0.05, ^**^ *p* < 0.01, compared with WT group; ^#^ *p* < 0.05, ^##^ *p* < 0.01, compared with APP/PS1 group.

In this study, a significant downward trend ((*p* < 0.01)) of CA and CDCA was also found in APP/PS1 model mice (Figures 9A,B). After 3 months of AOF administration, CA and CDCA levels of APP/PS1 mice showed an upward (*p* < 0.05) trend. Conjugated primary bile acids TCA (Figure 9C, *p* < 0.05), GCA (Figure 9D, *p* < 0.05), TCDCA (Figure 9E, *p* < 0.01), GCDCA (Figure 9F, *p* < 0.01) had an upward trend in APP/PS1 group. After 3 months of AOF treatment, the metabolic disorders of TCA, GCA, TCDCA, and GCDCA were all alleviated to different degrees. Similarly, secondary bile acids DCA (Figure 9G, *p* < 0.05), LCA (Figure 9I, *p* < 0.01), and correspondingly conjugated bile acids GDCA (Figure 9K, *p* < 0.01), TDCA (Figure 9J, *p* < 0.01), TLCA (Figure 9N, *p* < 0.01), GLCA (Figure 9O, *p* < 0.05) were elevated in the APP/PS1 group and declined in the APP/PS1+AOF group. Notably, the metabolic performance of DCA and GCA in different groups was consistent with the predictions of untargeted metabolomics, validating the correctness of untargeted metabolomics.

**FIGURE 10:**
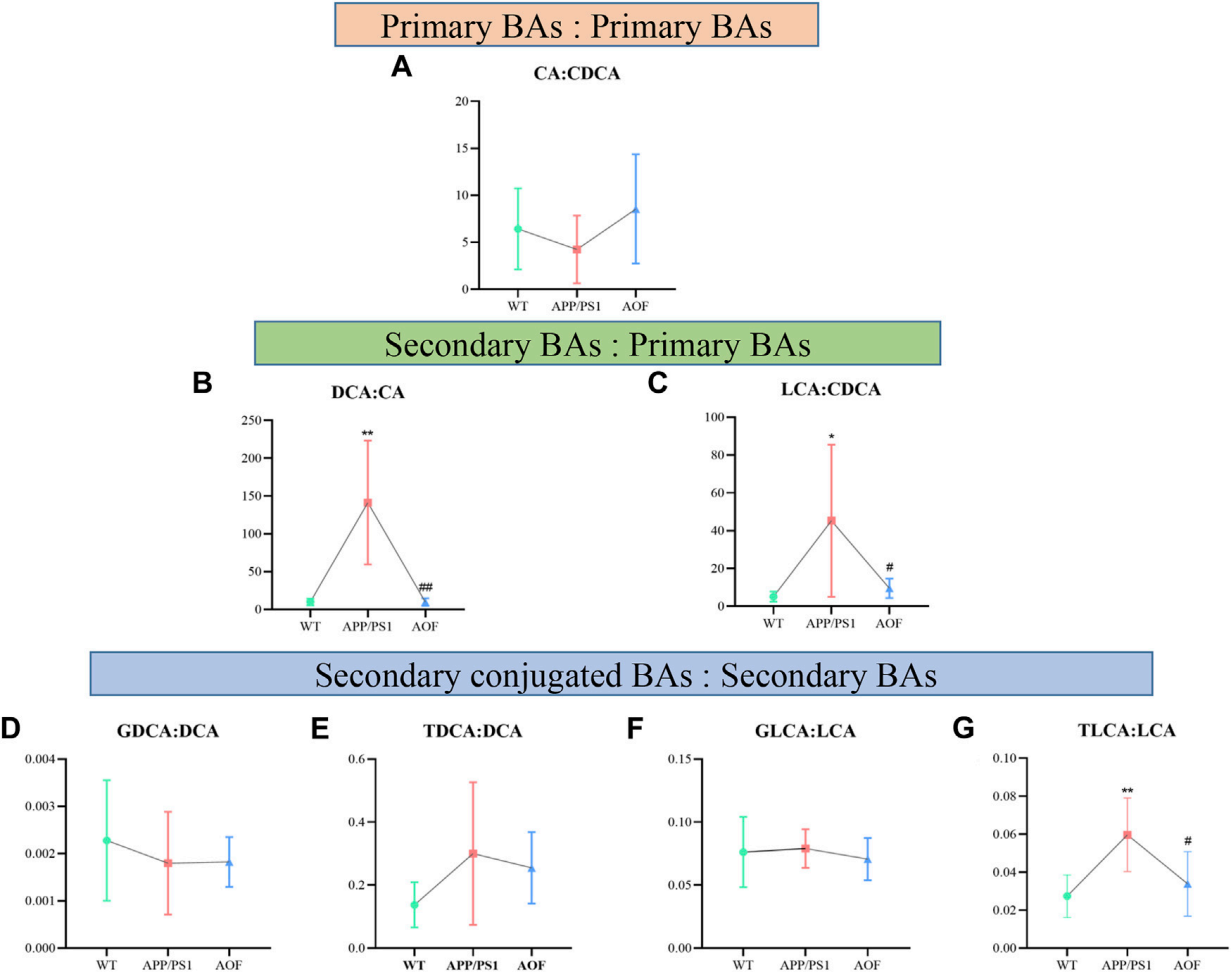
Ratios of primary and secondary BAs in different groups of APP/PS1 mice. **(A–G)** CA: CDCA, DCA: CA, LCA: CDCA, GDCA: DCA, TDCA: DCA, GLCA: LCA, and TLCA: LCA, respectively. Data are presented as mean ± SEM (*n* = 6). ^*^ *p* < 0.05, ^**^ *p* < 0.01, compared with WT group; ^#^ *p* < 0.05, ^##^ *p* < 0.01, compared with APP/PS1 group.

To explore which enzymatic processes play a key role in bile acid metabolism, we explored seven bile acid ratios in different groups of mice (Figure 10). The ratios of primary BAs to primary BAs (CA: CDCA) can reflect changes in the classical and alternative metabolic pathways of liver BAs (MahmoudianDehkordi et al., 2019). As shown in Figure 10A, CA: CDCA ratio had no significant change in the WT group and APP/PS1 group, and the callback effect was not obvious after AOF treatment. DCA: CA and LCA: CDCA is a common form of secondary bile acids: primary bile acids that reflect changes in gut microbiome enzymes. Among them, DCA: CA is significantly related to the diagnosis of human Alzheimer’s disease (Nho et al., 2019). In this study, significant differences in DCA: CA (Figure 10B) and LCA: CDCA (Figure 10C) values were found in plasma samples from different groups of mice. AOF can reduce DCA: CA and LCA: CDCA values in APP/PS1 mice, suggesting that AOF may slow down the progression of AD from the bile acid metabolism pathway. Finally, we investigated the effect of the combination of taurine and glycine on the dysmetabolism of secondary bile acids (Figures 10D–G). Except for TLCA: LCA, GDCA: DCA, TDCA: DCA, and GLCA: LCA had no significant association with AD, and AOF failed to significantly alter the metabolic disorder of bile acids, although there was a trend of correction.

## 4 Discussion

This research was the first to confirm the anti-AD effect of AOF and explore its potential mechanism by combining the overall behavioral performance, brain plaque deposition, plasma untargeted metabolomics, and targeted metabolomics results of transgenic mice. In this study, UHPLC-Q-Orbitrap HRMS was utilized to qualitatively analyze AOF, and 20 sesquiterpenoids were identified. As a characteristic component of AOF, sesquiterpenoids had been shown to play a neuroprotective role by alleviating oxidative stress, and have significant effects on amyloid-β-triggered cognitive impairment and neuronal abnormalities (Shi et al., 2014; Zhang et al., 2018). In addition, kaempferol (Li et al., 2019), 5-HMF (Liu et al., 2014), and chrysin (Talebi et al., 2021) had been proved to have anti-AD potential. The above studies on the material basis of AOF suggested the therapeutic potential and value of AOF for AD.

The classic pathological feature of AD is the abnormal deposition of Aβ in the brain forming a large number of Aβ plaques (Ballard et al., 2011). Mutations in APP and PS1 genes lead to excessive deposition of A*β*_42_ for the purpose of simulating the pathological features of AD (Vassar, 2004), and therefore APP/PS1 mode is recognized worldwide as a transgenic animal model for exploring AD. MWM, the gold standard of AD behavior, is commonly utilized to test the memory and learning ability of rodents. The MWM experiment demonstrated that AOF could improve the learning and memory impairment of APP/PS1 model mice. IHC was used to compare the expression levels of Aβ in the hippocampus and cortex of different groups of mice. The yellow-brown plaques in the APP/PS1 group were considerably more than in other groups. After AOF treatment, the plaques became less numerous and lighter in color. These results suggested that AOF may treat AD by reducing the expression of Aβ. In conclusion, AOF could adjust the behavioral characteristics and reduced the abnormal accumulation of Aβ in the hippocampus and cortex, which provided an experimental basis for the clinical use of AOF in the therapy of AD.

The etiology of AD is complex, and AOF has been commonly used to treat forgetfulness since ancient times. This study attempted to explain the therapeutic effect of AOF in terms of the disturbance and back regulation of potential differential metabolites in plasma. 31 plasma differential metabolites were found between the APP/PS1 and WT groups, 15 of which could be significantly back-regulated by AOF. It mainly involved sphingolipid metabolism, TCA cycle, glycerophospholipid metabolism, tyrosine metabolism, and primary bile acid biosynthesis. Lipids are critical components for maintaining normal cell structure and function, storing energy, and conducting signals (Gaschler and Stockwell, 2017; Wong et al., 2017). Dysregulation of lipid homeostasis is highly associated with neurodegenerative disease AD (Kao et al., 2020). In our study, AOF might improve AD symptoms through sphingolipid, glycerophospholipid, fatty acid, and bile acid metabolic pathways.

Sphinganine (Sa), sphinganine-1-phosphate (Sa1P), sphingosine-1-phosphate (S1P), ceramide (Cer), and phytosphingosine located in the sphingolipid metabolism pathway were all differential metabolites of WT and APP/PS1 groups. Compared with the WT group, Sa, Sa1P, and S1P levels in APP/PS1 group were significantly upregulated. After 3 months of AOF treatment, Sa1P showed a downward trend, and Sa and S1P considerably decreased. Ceramides are sphingolipids with a wide range of biological activities (Di Pardo and Maglione, 2018). Our data showed that the levels of phytosphingosine and cer (d18:0/14:0) in the APP/PS1 group decreased significantly, and the concentration of cer (d18:0/14:0) showed an upward trend after AOF treatment. Previous studies have shown that glycerophospholipid metabolism is closely related to the pathogenesis of AD (Kalli, 2020). Phosphatidylcholine (PC), phosphatidylethanolamine (PE), lysoPC, and lysoPE were located in glycerophospholipid metabolism. It found that increased lysoPC intensifies Aβ deposition and induces neurotoxicity (Sheikh et al., 2015). In our study, lysoPC(17:0), lysoPE (20:2), and lysoPE (22:6) levels increased observably in the APP/PS1 group and decreased markedly after AOF treatment. In the fatty acid metabolic pathway, the levels of palmitic acid, stearic acid, docosahexaenoic acid (DHA), and eicosapentanoic acid (EPA) all prominently escalate in APP/PS1 groups. After AOF treatment, palmitic acid, stearic acid, and EPA showed a downward trend, while DHA was significantly improved. Palmitic acid and stearic acid have been proven to increase amyloid protein in cortical neurons and promote tau hyperphosphorylation. DHA (Bazan et al., 2011; Petermann et al., 2022) and EPA (Shahidi, 2018) are unique polyunsaturated fatty acids, which can regulate synaptic function and improve salient transmission, and have great significance in neurodegenerative diseases.

BAs eliminate liver cholesterol, a crucial factor in AD. In the liver, cholesterol is converted into the primary bile acids CDCA and CA. Secondary bile acids (DCA, UDCA, LCA) are produced by the dehydrogenation of primary bile acids by 7α-dihydroxylation. AOF might improve AD symptoms because it could regulate probiotics and inhibit pathogenic bacteria. The link between the brain and the gut might be liaised by BAs metabolism.

## 5 Conclusion

AD is a complex neurodegenerative disease. In this work, we come to three conclusions. 1) AOF could correct the behavior of model mice and reduce the deposition of AB in the brain. 2) Untargeted metabolomics showed that 15 plasma metabolites were significantly back-regulated by AOF, mainly involving the sphingolipid, glycerophospholipid, fatty acid, and bile acid metabolic pathways. 3) After AOF treatment, all BAs showed a correction trend, and most of the metabolites went back significantly. In summary, our study explored the mechanism of action of AOF in treating AD, and provided evidence for BAs as a potential diagnostic indicator for AD.

## Data Availability

The original contributions presented in the study are included in the article/Supplementary Material, further inquiries can be directed to the corresponding author.
