# Supplementary material for: Integrated untargeted and targeted metabolomics to reveal therapeutic effect and mechanism of Alpiniae oxyphyllae fructus on Alzheimer’s disease in APP/PS1 mice
Source: Front Pharmacol. 2023 Jan 11;13:1104954. doi: 10.3389/fphar.2022.1104954 (PMC9873993; doi:10.3389/fphar.2022.1104954)
Supplement: Supplementary file 1 [file Table1.docx]

**Supplementary Table S1** The mass spectrum information of 41 identified compounds in *Alpiniae Oxyphyllae* Fructus.

| **No.** | **Identification** | **Formula** | **Ion mode** | **t_R_/**  **min** | **Calculated**  **MS (*m/z*)** | **Experimental**  **MS (*m/z*)** | **Error**  **(ppm)** | **Fragments ions (*m/z*)** | **Type** |
| --- | --- | --- | --- | --- | --- | --- | --- | --- | --- |
| 1 | Betaine | C_5_H_11_NO_2_ | [M+H]^+^ | 1.29 | 118.08 625 | 118.08 620 | -0.467 | 72.08,58.07 | Alkaloids |
| 2 | 5-hydroxymethylfurfural | C_6_H_6_O_3_ | [M+H]^+^ | 2.73 | 127.03 897 | 127.03 887 | -0.792 | 127.04,109.03,81.03,53.04 | Aldehydes |
| 3 | Protocatechuic acid | C_7_H_6_O_4_ | [M-H]^-^ | 3.33 | 153.01 933 | 153.01 913 | 5.848 | 153.02,109.03,91.02,81.03 | Phenolic acids |
| 4 | Protocatechuic aldehyde | C_7_H_6_O_3_ | [M+H]^+^ | 5.52 | 139.03 897 | 139.03 902 | 0.355 | 139.04,111.04,93.03 | Aldehydes |
| 5 | Chrysin | C_15_H_10_O_4_ | [M+H]^+^ | 5.6 | 253.05 063 | 253.04 979 | 1.007 | 225.06,209.06,151.00,107.01,101.04 | Flavonoids |
| 6 | Vanillic acid | C_8_H_8_O_4_ | [M-H]^-^ | 6.53 | 167.03 498 | 167.03 377 | -0.69 | 152.01,123.04,108.02 | Phenolic acids |
| 7 | Oxyphyllanene A | C_12_H_16_O_2_ | [M+H]^+^ | 7.18 | 193.12 230 | 193.12 225 | -0.292 | 193.12,175.11,165.13,147.12,123.08 | Eudesmane sesquiterpenoids |
| 8 | Yakuchinone B | C_20_H_22_O_3_ | [M+H]^+^ | 8.7 | 311.16 417 | 311.16 348 | -2.221 | 293.15,233.12,177.05,137.06 | Diarylheptanoids |
| 9 | Teuhetenone A | C_12_H_18_O_2_ | [M+H]^+^ | 9.36 | 195.13 795 | 195.13 770 | -2.577 | 177.13,159.12,149.13,121.07 | Eudesmane sesquiterpenoids |
| 10 | Oxyphyllenone A | C_12_H_18_O_3_ | [M+H]^+^ | 10.22 | 211.13 287 | 211.13 274 | -0.62 | 193.12,175.11,151.11,133.10 | Eudesmane sesquiterpenoids |
| 11 | Kaempferol | C_15_H_10_O_6_ | [M+H]^+^ | 10.66 | 287.05 501 | 287.05 441 | -2.106 | 287.05,269.04,241.05,179.03 | Flavonoids |
| 12 | Perillaldehyde | C_10_H_14_O | [M+H]^+^ | 11.14 | 151.11 174 | 151.11 153 | -1.401 | 133.10,123.12,109.10,81.07 | Aldehydes |
| 13 | Isovanilline | C_8_H_8_O_3_ | [M+H]^+^ | 12.31 | 153.05 462 | 153.05 452 | -0.658 | 153.05,125.06,111.04 | Aldehydes |
| 14 | Oxyphyllenone B | C_12_H_18_O_3_ | [M+H]^+^ | 13.68 | 211.13 287 | 211.13 281 | -0.289 | 193.12,175.11,151.11,133.10 | Eudesmane sesquiterpenoids |
| 15 | Baicalein | C_15_H_10_O_5_ | [M+H]^+^ | 13.84 | 271.06 009 | 271.05 957 | -1.955 | 271.06,243.07,163.04 | Flavonoids |
| 16 | Rhamnocitrin | C_16_H_12_O_6_ | [M+H]^+^ | 14.8 | 301.07 066 | 301.07 010 | -1.875 | 286.05,258.05 | Flavonoids |
| 17 | kaemperol-4'-methylester | C_16_H_12_O_6_ | [M-H]^-^ | 14.87 | 299.05 611 | 299.05 557 | 1.857 | 284.03,271.06,243.07,228.04,212.05 | Flavonoids |
| 18 | Tectochrysin | C_16_H_12_O_4_ | [M+H]^+^ | 18.25 | 269.08 083 | 269.08 035 | -1.804 | 269.08,254.06,226.06,167.03,124.02 | Flavonoids |
| 19 | Methyl ferulate | C_12_H_14_O_4_ | [M-H]^-^ | 18.28 | 221.08 193 | 221.08 112 | -3.674 | 177.09,149.10,121.03,93.03 | Others |
| 20 | Oxyphyllenodiol A | C_14_H_22_O_3_ | [M+H]^+^ | 19.08 | 239.16 417 | 239.16 394 | -0.966 | 221.15,203.14,193.16,175.15,145.10 | Cadinane sesquiterpenoids |
| 21 | Oxyphyllanene B | C_12_H_14_O_2_ | [M+H]^+^ | 21.24 | 191.10 665 | 191.10 666 | 0.02 | 191.11,173.10,163.11,145.10,135.08 | Eudesmane sesquiterpenoids |
| 22 | 7-Epi-teucrenone | C_15_H_22_O_2_ | [M+H]^+^ | 21.82 | 235.16 925 | 235.16 899 | -1.133 | 217.16,199.15,189.16,149.10 | Eudesmane sesquiterpenoids |
| 23 | 7a(H),10β-eudesm-4-en-3-one-11,12-diol | C_15_H_24_O_3_ | [M+H]^+^ | 21.84 | 253.17 982 | 253.17 944 | -1.505 | 235.17,217.16,177.13 | Eremophilane sesquiterpenoids |
| 24 | Pinene | C_10_H_16_ | [M+H]^+^ | 22.6 | 137.13 247 | 137.13 237 | -0.781 | 95.09,81.07,67.06 | Others |
| 25 | 4S-isoprophy-6-methyl-1-tetralone | C_14_H_18_O | [M+H]^+^ | 23.48 | 203.14 304 | 203.14 299 | -0.255 | 185.13,175.15,159.12,133.10,119.09,  105.07 | Cadinane sesquiterpenoids |
| 26 | Oxyphyllanene F | C_15_H_22_O_3_ | [M+H]^+^ | 26.22 | 251.16 417 | 251.16 409 | -0.323 | 233.15,215.14,205.16,191.14,149.10 | Eudesmane sesquiterpenoids |
| 27 | Oxyphyllenodiol B | C_14_H_22_O_3_ | [M+H]^+^ | 28.02 | 239.16 417 | 239.16 379 | -1.593 | 221.15,203.14,193.16,175.15,145.10 | Cadinane sesquiterpenoids |
| 28 | Oxyphyllol B | C_15_H_22_O_2_ | [M+H]^+^ | 29.95 | 235.16 925 | 235.16 916 | -0.41 | 217.16,189.16,175.11,161.10,149.10 | Eremophilane sesquiterpenoids |
| 29 | Oplopanone* | C_15_H_26_O_2_ | [M+H]^+^ | 30.31 | 239.20 550 | 239.20 045 | -0.446 | 239.20,203.18,175.15,161.13,147.12 | Oplopanone sesquiterpenoids |
| 30 | Cymol | C_10_H_14_ | [M-H]^-^ | 33.26 | 133.10 227 | 133.10 112 | -0.428 | 133.10,117.09,105.07 | Others |
| 31 | Cryptotanshinone | C_19_H_20_O_3_ | [M+H]^+^ | 33.08 | 297.14852 | 297.14804 | -1.619 | 282.13,279.14,268.11,253.09,251.14 | Others |
| 32 | 11-Hydroxy-valenc-1(10)-en-2-one | C_15_H_24_O_2_ | [M+H]^+^ | 34.70 | 237.18 490 | 237.18 468 | -0.955 | 219.17,201.16,191.18 | Eremophilane sesquiterpenoids |
| 33 | Izalpinin | C_16_H_12_O_5_ | [M+H]^+^ | 35.02 | 285.07 575 | 285.07 529 | -1.614 | 270.05,242.06,167.03,118.04 | Flavonoids |
| 34 | Nootkatone* | C_15_H_22_O | [M+H]^+^ | 35.72 | 219.17 434 | 219.17 430 | -0.191 | 201.16,191.18,163.11,149.10 | Eremophilane sesquiterpenoids |
| 35 | dehydro-nootkatone | C_15_H_20_O | [M+H]^+^ | 39.47 | 217.15 869 | 217.15 854 | -0.699 | 199.15,189.16,175.11,161.10,133.10 | Eremophilane sesquiterpenoids |
| 36 | β-stigmasterol | C_29_H_48_O | [M+H]^+^ | 40.13 | 413.37 779 | 413.37 704 | -1.821 | 413.38,395.37,123.08,109.07 | Sterols |
| 37 | Oleanic acid | C_30_H_48_O_3_ | [M+H]^+^ | 40.16 | 457.36 762 | 457.36 664 | -2.147 | 457.37,439.36,411.36 | Others |
| 38 | Oxyphyllenone H | C_14_H_22_O_2_ | [M+H]^+^ | 42.78 | 223.16 925 | 223.16 904 | -0.97 | 205.16,187.15,163.11,145.10,119.09 | Cadinane sesquiterpenoids |
| 39 | Valencene* | C_15_H_24_ | [M+H]^+^ | 44.12 | 205.19 507 | 205.19 479 | -1.4 | 205.20,163.15,149.13,135.12,121.10 | Eremophilane sesquiterpenoids |
| 40 | Eremophila-1(10),11(12)-diene-2,9-dione | C_15_H_20_O_2_ | [M+H]^+^ | 45.27 | 233.15 360 | 233.15 341 | -0.842 | 215.14,205.16,191.11,137.10,109.10 | Eremophilane sesquiterpenoids |
| 41 | Oxyphyllone E | C_14_H_20_O_3_ | [M-H]^-^ | 45.7 | 235.13 396 | 235.13 379 | 0.919 | 235.13,217.12,191.14 | Cadinane sesquiterpenoids |

*Compared with standard substance

**Supplementary Table S2** MRM transitions, retention time and collision energy level for the 15 bile acids that were quantified in plasma samples.

| Analytes | Precursor  ion (M−H) ^−^ | Product  ion | Retention  time (min) | DP  (volts) | EP  (volts) | CXP  (volts) |
| --- | --- | --- | --- | --- | --- | --- |
| Cholic acid (CA) | 407.30 | 407.30 | 1.696 | -80.0 | -11.0 | -4.0 |
| Deoxycholic acid (DCA) | 391.30 | 391.30 | 2.512 | -70.0 | -8.0 | -3.0 |
| Chenodeoxycholic acid (CDCA) | 391.31 | 391.31 | 2.270 | -90.0 | -10.0 | -6.0 |
| Ursodiol (UDCA) | 391.32 | 391.32 | 1.586 | -90.0 | -10.0 | -4.0 |
| Lithocholic acid (LCA) | 375.30 | 375.30 | 3.445 | -75.7 | -7.6 | -3.6 |
| Glycocholic acid (GCA) | 464.30 | 464.30 | 1.800 | -95.0 | -10.0 | -6.0 |
| Glycolithocholic acid (GLCA) | 432.30 | 432.30 | 3.647 | -80.0 | -10.0 | -6.0 |
| Glycodeoxycholic acid (GDCA) | 448.30 | 448.30 | 2.738 | -90.0 | -9.0 | -6.0 |
| Glycochenodeoxycholic acid (GCDCA) | 448.31 | 448.31 | 2.506 | -90.0 | -9.0 | -6.0 |
| Glycoursodeoxycholic acid (GUDCA) | 448.32 | 448.32 | 1.701 | -90.0 | -9.0 | -6.0 |
| Taurocholic Acid (TCA) | 514.30 | 514.30 | 1.911 | -128.0 | -10.0 | -8.0 |
| Taurolithocholic acid (TLCA) | 482.30 | 482.30 | 3.900 | -102.0 | -10.0 | -8.0 |
| Taurodeoxycholic acid (TDCA) | 498.30 | 498.30 | 2.944 | -115.0 | -10.0 | -8.0 |
| Taurochenodeoxycholic acid (TCDCA) | 498.31 | 498.31 | 2.744 | -115.0 | -10.0 | -8.0 |
| Tauroursodeoxycholic acid (TUDCA) | 498.32 | 498.32 | 1.81 | -115.0 | -10.0 | -8.0 |
