# Supplementary figures and images for: Integrated untargeted and targeted metabolomics to reveal therapeutic effect and mechanism of Alpiniae oxyphyllae fructus on Alzheimer’s disease in APP/PS1 mice
Source: Front Pharmacol. 2023 Jan 11;13:1104954. doi: 10.3389/fphar.2022.1104954 (PMC9873993; doi:10.3389/fphar.2022.1104954)

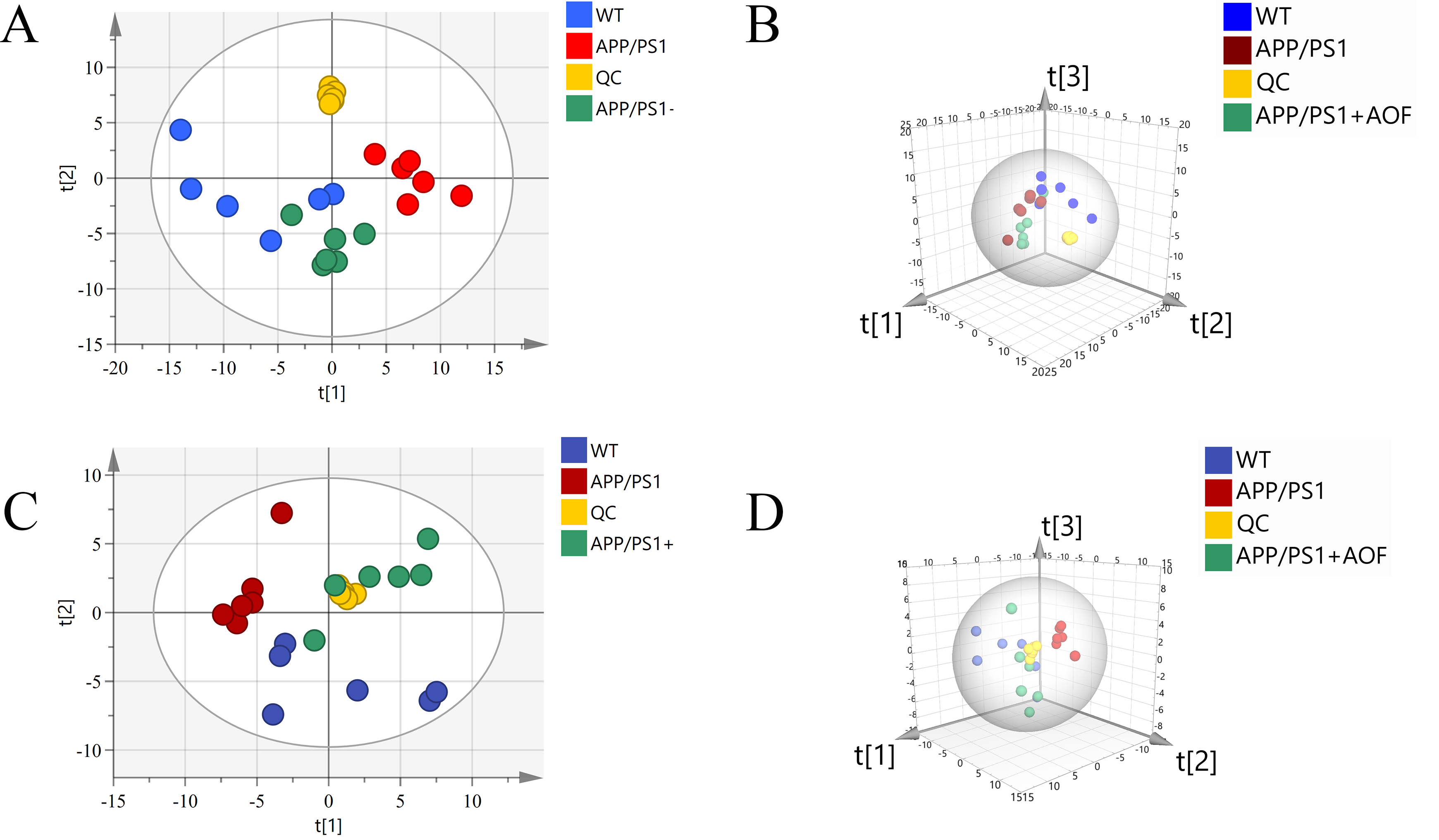

Supplement: Supplementary file 2 [file Image2.tif]

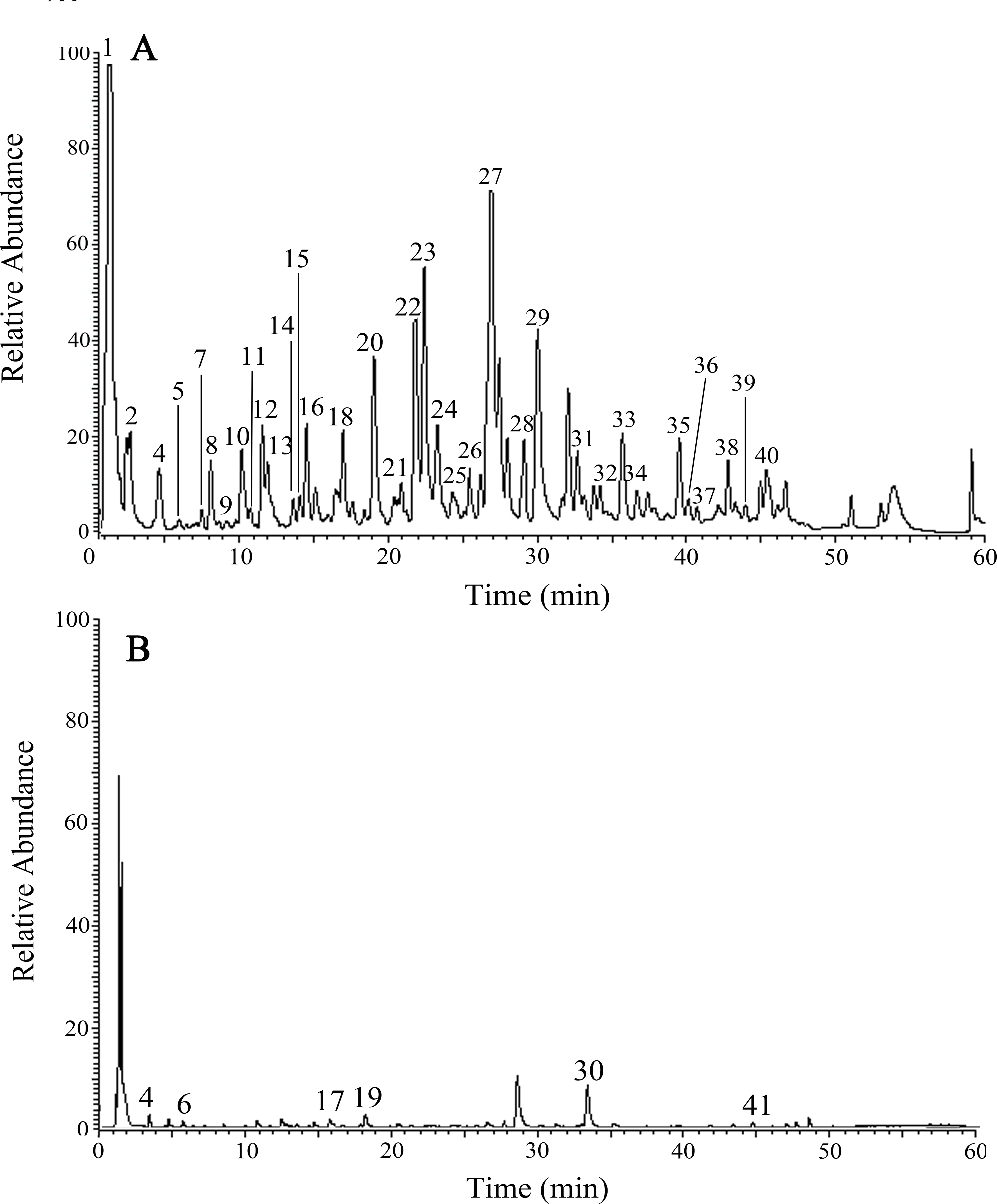

Supplement: Supplementary file 3 [file Image1.tif]
